# Automatic ultrasound image alignment for diagnosis of pediatric distal forearm fractures

**DOI:** 10.1007/s11548-025-03361-w

**Published:** 2025-05-02

**Authors:** Peng Liu, Yujia Hu, Jurek Schultz, Jinjing Xu, Christoph von Schrottenberg, Philipp Schwerk, Josephine Pohl, Guido Fitze, Stefanie Speidel, Micha Pfeiffer

**Affiliations:** 1 Translational Surgical Oncology, National Center for Tumor Diseases, Dresden, Germany; 2 German Cancer Research Center (DKFZ), Heidelberg, Germany; 3 Faculty of Medicine and University Hospital Carl Gustav Carus, University of Technology Dresden, Dresden, Germany; 4 Helmholtz-Zentrum Dresden-Rossendorf (HZDR), Dresden, Germany; 5 Centre for Tactile Internet with Human-in-the-Loop, University of Technology Dresden, Dresden, Germany; 6 Department of Pediatric Surgery, Faculty of Medicine and University Hospital Carl Gustav Carus, University of Technology Dresden, Dresden, Germany

**Keywords:** Ultrasound, Alignment, Pediatric, Fracture

## Abstract

****Purpose**:**

The study aims to develop an automatic method to align ultrasound images of the distal forearm for diagnosing pediatric fractures. This approach seeks to bypass the reliance on X-rays for fracture diagnosis, thereby minimizing radiation exposure and making the process less painful, as well as creating a more child-friendly diagnostic pathway.

****Methods**:**

We present a fully automatic pipeline to align paired POCUS images. We first leverage a deep learning model to delineate bone boundaries, from which we obtain key anatomical landmarks. These landmarks are finally used to guide the optimization-based alignment process, for which we propose three optimization constraints: aligning specific points, ensuring parallel orientation of the bone segments, and matching the bone widths.

****Results**:**

The method demonstrated high alignment accuracy compared to reference X-rays in terms of boundary distances. A morphology experiment including fracture classification and angulation measurement presents comparable performance when based on the merged ultrasound images and conventional X-rays, justifying the effectiveness of our method in these cases.

****Conclusions**:**

The study introduced an effective and fully automatic pipeline for aligning ultrasound images, showing potential to replace X-rays for diagnosing pediatric distal forearm fractures. Initial tests show that surgeons find many of our results sufficient for diagnosis. Future work will focus on increasing dataset size to improve diagnostic accuracy and reliability.

**Supplementary Information:**

The online version contains supplementary material available at 10.1007/s11548-025-03361-w.

## Introduction

Distal forearm fractures are the most prevalent type of fractures, accounting for 29% of all fractures in children aged 0–15 years [1, 2]. This high frequency highlights the importance of establishing a reliable diagnostic pathway to replace traditional $\text {X-rays}$, aiming to accurately detect distal forearm fractures while minimizing radiation exposure to the lowest possible levels. Point-of-care ultrasound (POCUS) of the wrist is a radiation-free, quick and user-friendly imaging alternative. Moreover, POCUS allows children to stay with their parents, which helps avoid the separation often required for radiation safety. Additionally, POCUS may be less uncomfortable since it eliminates the need to position the arm for X-rays [3, 4].

**Fig. 1:**
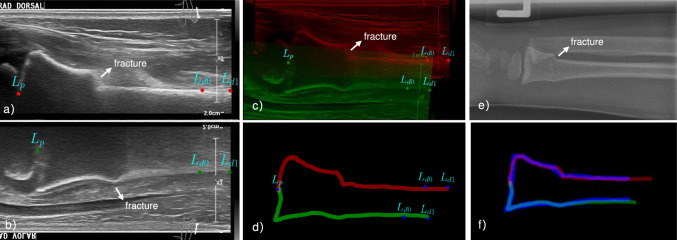
POCUS and X-ray images of a 6-year-old patient with a metaphyseal, dorsally angulated radius fracture (buckle fracture). **a** Dorsal POCUS image of radius bone, red points are landmarks. **b** Flipped palmar POCUS image of radius bone, green points are landmarks. **c** aligned dorsal (red) and palmar (green) image and landmarks. **d** Aligned segmented boundaries and landmarks. **e** reference lateral X-ray image. **f** Registered POCUS boundaries (red and green) with X-ray boundary (blue)

During POCUS, the radius and ulna are systematically imaged in six longitudinal planes: three for the radius (dorsal, lateral, and palmar) and three for the ulna (palmar, medial, and dorsal)[3]. These images fundamentally differ from traditional $\text {X-rays}$, which produce a shadow-like image of all bony structures in either a lateral or anterior–posterior plane. POCUS reliably detects distal forearm fractures in pediatric and adolescent patients with partially open physes, especially non-articular fractures of the distal radius. However, excellent diagnostic sensitivity and specificity have only been published for detecting any fracture or identifying specific types of fractures, not for their exact description, including quantification of displacement or angulation.

Diagnosing these fractures accurately can be challenging for physicians accustomed to X-rays. Standard POCUS guidelines recommend X-rays if an angulated fracture is suspected [4]. Eliminating this need could make distal forearm fracture diagnosis and management entirely X-ray-independent. A potential solution for visualizing fractured bone angulation is merging volar and dorsal POCUS views. Aligning these images—ensuring parallel bone-soft-tissue interfaces in the diaphysis and aligned physes—creates a lateral X-ray-like view without ulna superposition (see c in Fig. 1). This allows for precise angulation quantification and physeal fracture displacement estimation, similar to conventional lateral X-rays [5–7]. These alignments can be done manually [3], but the process is tedious and time-consuming, making it impractical for routine emergency care. To address this issue, we propose an automatic pipeline for aligning POCUS views to replace X-rays for fracture diagnosis, which is the pioneering work in this field to our best knowledge.

## Methods

Given two POCUS images $\textbf{I}_f$ and $\textbf{I}_m$ of the same bone of children’s forearm from two opposite orientations, such as dorsal and palmar images (see a and b in Fig 1), the goal is to find the optimal transformation matrix $\mathcal {T}\in \textrm{R}^{3 \times 3}$ so that $\mathcal {T}(\textbf{I}_m)$ is aligned with the fixed $\textbf{I}_f$ in an anatomically correct view. We define this alignment task as an optimization problem with the assistance of sparse landmarks $\{{L^f, L^m}\}$ on both POCUS images.

### Landmark extraction

The extracted landmarks are required to represent the rough contour of bone and can be employed for the subsequent alignment. Due to the high noise levels and limited structural features available in POCUS images, exploring such qualified sparse landmarks directly and automatically is challenging. To address this, we reformulate the task as a segmentation problem as first step, where we reformulate nnUnet [8] to delineate the prominent boundary of the bones (see d in Fig 1). From the segmentation results, three landmark candidates are derived based on morphological and physic properties of the bone: $L_p$ represents the position of physis, typically located at the lower-left region of the segmented boundary, $L_{d0}$ and $L_{d1}$ correspond to the diaphysis, where $L_{d1}$ is identified in the right part of the long horizontal segment, and $L_{d0}$ is approximately 2–3 cm away from $L_{d1}$. Delineating the shape of the radius bone, three feature points $L^{f}=\{L_{p}^{f}, L_{d0}^{f}, L_{d1}^{f}\}$ and $L^{m}=\{L_{p}^{m}, L_{d0}^{m}, L_{d1}^{m}\}$ are automatically identified for both POCUS images. In practice, these three feature points have proven sufficient for the subsequent pose estimation step.

### Pose estimation

Given the limited overlap and correspondence information between POCUS views, the alignment process relies on defining constraints based on the anatomical features of the bone. By fixing $I_F$ and $L_F$, we propose three constraints to minimize, integrated into the pose estimation optimization target:

*Latitudinal constraint*($C_{a}$): The two physis points from each view should align in such a way that their components on the X-axis should be close to each other and their Y-components should be within a specific range *d*. Constraint $C_{a}$ is defined as a weighted sum of the two distances:

1 $$\begin{aligned} C_{a}=w_x \vert L_{p,x}^f - \mathcal {T}(L_{p,x}^m) \vert + w_y \vert d - L_{p,y}^f + \mathcal {T}(L_{p,y}^m) \vert \nonumber \\ \end{aligned}$$

*Longitudinal orientation constraint*($C_{o}$): The transformation is constrained by unit orientation vectors of diaphysis $\textbf{i}_d^f=(L_{d0}^f - L_{d1}^f)/\Vert L_{d0}^f - L_{d1}^f \Vert$ and $\textbf{i}_d^m=(\mathcal {T}(L_{d0}^m - L_{d1}^m))/\Vert L_{d0}^m - L_{d1}^m \Vert$ which should be parallel to each other:

2 $$\begin{aligned} C_{o}=\Vert \textbf{i}_d^f - \textbf{i}_d^m \Vert \end{aligned}$$

*Longitudinal position constraint*($C_{p}$): The first two constraints can fall into local minima on their own, for example, all diaphysis points could be placed in one straight line. Therefore, the distance between the line segments formed by diaphysis landmarks on the moving image and the ones of the fixed image should equal the bone width:

3 $$\begin{aligned} C_{p}=\vert (\mathcal {T}(L_{d,mid}^m) - L_{d0}^f) \cdot \textbf{n}^f \vert \end{aligned}$$

where $L_{d,mid}^m$ is the middle point of diaphysis landmarks of the moving image and $\textbf{n}^f$ is the normal vector of $\textbf{i}_d^f$.

4 $$\begin{aligned} E(L^f, L^m, \mathcal {T})=w_{a}C_{a} + w_{o}C_{o} + w_{p}C_{p} \end{aligned}$$

The overall optimization target function combines above constraints as a weighted sum as Eq 4. To minimize the target, we employed the Gradient Descent algorithm with a learning rate $5 e^{-2}$ and 10,000 iterations to iteratively align the two POCUS images.

## Evaluation

In this section, we present two experiments: alignment experiment and morphology experiment, the former is designed to show the accuracy of the proposed aligning approach, while the latter is carried out to show the effectiveness of diagnosing distal forearm fracture with the merged POCUS images. All the experiments are conducted on radius bone images from dorsal and palmar views since the radius bone is more often fractured and more important for clinical diagnostic decisions compared to ulna bone. Also, ulna POCUS images can be merged following the same strategy the same groups of landmarks can be extracted. The successful alignment of radius bone images demonstrated the ability to align the ulna bone and other scanning orientations with our pipeline. All the experiments were performed on computer with Xeon Silver 4216 CPU and Nvidia RTXA5000 GPU.

For training nnUnet, boundaries of 40 images from 20 patients are segmented manually by brush tools with a width of around 1 mm on CVAT platform (http://www.cvat.ai), by technical staff and checked by the surgeons. The 20 patients consist of healthy patients and patients with buckle fracture and greenstick fracture. The 40 images are then split into a training dataset with 36 images, and a test dataset the 4 images. The trained nnUnet achieved a 0.78 Dice score on the test dataset.

### Alignment experiment

We evaluate the performance of the pose estimation approach by registering the aligned POCUS images $\textbf{I}_f~\text {and}~ \textbf{I}_m$ to the reference lateral X-ray images $\textbf{I}_r$ with distal radius bone segmented (see f in Fig 1). This process is done manually on 10 samples with ${\textbf{I}_f, \textbf{I}_m, \textbf{I}_r}$, which are unseen during training. Directed Hausdorff distance and Chamfer distance are calculated between the registered segmentations, resulting in 2.66 mm and 1.05 mm mean registration errors, respectively. Qualitatively, the aligned ultrasound segmentations depict the same shape as the reference boundary in X-rays. As confirmed by the clinician, the aligned POCUS images contain all the necessary information for diagnosis of distal forearm fracture.

### Morphology experiment

**Fig. 2:**
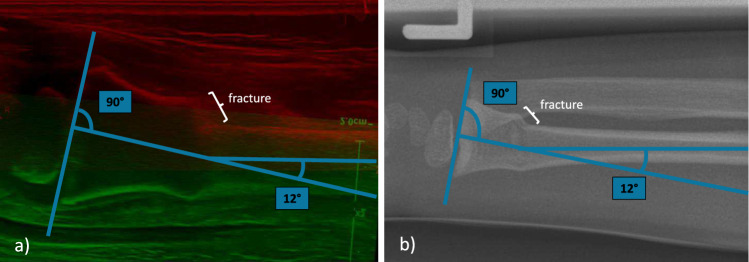
**a** Aligned POCUS images of a distal forearm of a 6-year-old patient with a metaphyseal, dorsally angulated radius fracture (buckle fracture). **b** Corresponding lateral radiograph with a metaphyseal, dorsally angulated radius fracture. The degree of dislocation ($12^{\circ }$) is annotated in both images with the perpendicular line to the growth plate being the reference

**Table 1:** Labeled types of fractures (B=buckle fracture, G=greenstick fracture, and N=no fracture) using merged POCUS images and X-ray images of 11 samples by three participants. The ground-truth (GT) types are the results of majority voting of all the labels from three participants. Correct predictions are colored in , and wrong ones in

Sample	Participant 1		Participant 2		Participant 3		GT
	POCUS	X-Ray	POCUS	X-Ray	POCUS	X-Ray	
Sample 0		B		B		N	B
Sample 1		N		N		N	N
Sample 2		N		N		N	N
Sample 3		G		G		G	G
Sample 4		B		B		B	B
Sample 5		B		B		B	B
Sample 6		N		N		N	N
Sample 7		B		B		B	B
Sample 8		N		N		N	N
Sample 9		B		B		B	B
Sample 10		B		B		B	B
Accuracy	0.82	1.0	0.82	1.0	0.64	0.91	/

To further evaluate the utility of the merged POCUS images for diagnosis of distal forearm fracture, we carried out a dual-modality morphology experiment comprising fracture classification and angulation measurement. Following a standardized measurement protocol, the dislocation angle of the distal fragment was determined by constructing a perpendicular reference line to the growth plate (see Fig. 2). This method is applied to both X-ray and POCUS images. The experiment dataset consists of 11 matched X-ray and POCUS images from the same patients. Three blinded evaluators including two pediatric traumatologists (participants 1 & 2) and one physician (participant 3) independently performed: (1) fracture classification following [3], (2) angulation measurement for two groups of images without knowing the correspondences.

The classification results (B=buckle fracture, G=greenstick fracture, and N=no fracture) from all three participants are listed in Table 1, with the ground-truth labels obtained by triple majority voting of X-ray labels. Consequently, diagnosis accuracy is introduced to show the performance of each participant on each group of images. Although the mean accuracy on merged POCUS images (0.76) is lower than the ones using X-ray (0.97), participants 1 and 2 obtained comparable accuracy on POCUS (0.82) as X-ray images, reflecting the consistency of expert knowledge. Notably, all participants misclassified sample 10 as buckle fracture, potentially due to a high amount of noise and artifact, and acquisition plane ambiguities.

**Table 2:** Measured angulation of merged POCUS images and X-ray images of 11 samples by three participants. The reference degrees are the mean degrees of the three participants. MAE refers to the mean absolute error of each column against reference values, and the no fracture samples are excluded. Bold faces refer to images with the lowest absolute error compared to reference, while the worst ones are underlined

**Sample**	Participant 1		Participant 2		Participant 3		References
	POCUS	X-Ray	POCUS	X-Ray	POCUS	X-Ray	
Sample 0	${\textbf {3.7}}^{\circ }$	$8.7^{\circ }$	$0.0^{\circ }$	$3.0^{\circ }$	–	–	$3.9^{\circ }$
Sample 1	–	–	–	–	–	–	–
Sample 2	–	–	–	–	–	–	–
Sample 3	$\underline{21.0}^{\circ }$	$10.0^{\circ }$	$\underline{20.0}^{\circ }$	$8.0^{\circ }$	$\underline{24.0}^{\circ }$	$8.0^{\circ }$	$8.7^{\circ }$
Sample 4	$7.9^{\circ }$	$15.0^{\circ }$	$9.0^{\circ }$	$8.0^{\circ }$	$17.0^{\circ }$	$5.0^{\circ }$	$9.3^{\circ }$
Sample 5	$9.1^{\circ }$	$7.0^{\circ }$	$4.0^{\circ }$	$2.0^{\circ }$	$10.0^{\circ }$	$7.0^{\circ }$	$5.3^{\circ }$
Sample 6	$3.0^{\circ }$	–	–	–	–	–	–
Sample 7	$4.2^{\circ }$	$9.3^{\circ }$	${\textbf {4.0}}^{\circ }$	$4.0^{\circ }$	${\textbf {7.0}}^{\circ }$	$9.0^{\circ }$	$7.3^{\circ }$
Sample 8	–	–	–	–	–	–	–
Sample 9	$5.8^{\circ }$	$9.7^{\circ }$	$8.0^{\circ }$	$10.0^{\circ }$	$10.0^{\circ }$	$9.0^{\circ }$	$9.6^{\circ }$
Sample 10	$4.2^{\circ }$	$14.3^{\circ }$	$6.0^{\circ }$	$8.0^{\circ }$	$8.0^{\circ }$	$9.0^{\circ }$	$10.4^{\circ }$
MAE	$4.4^{\circ }$ $\pm {3.7}^{\circ }$	$2.8^{\circ }$ $\pm {1.9}^{\circ }$	$3.7^{\circ }$ $\pm {3.4}^{\circ }$	$1.8^{\circ }$ $\pm {1.1}^{\circ }$	$5.0^{\circ }$ $\pm {4.9}^{\circ }$	$2.0^{\circ }$ $\pm {1.4}^{\circ }$	/

Angulation measurement results are presented in Table 2, where the reference angulation values are the mean over the three results measured with X-rays. For all images that are predicted as healthy, i.e., angulation is $0.0^{\circ }$, the angulation is denoted as "–". The mean absolute errors (MAEs) are calculated as the average of absolute difference between each measured angulations (merged POCUS or X-ray) and the reference angulations, and images with reference label being no fracture are excluded from the calculation of MAE. The resulting MAE with merged POCUS: $4.4^{\circ }$, $3.7^{\circ }$, and $5.0^{\circ }$, are larger than the X-ray counterparts being $2.8^{\circ }$, $1.8^{\circ }$, and $2.0^{\circ }$, but the discrepancy with $2.2^{\circ }$ in average suggests practical utility of aligned POCUS images for angulation assessment replacing X-rays. Furthermore, to show the influence of fractures on angulation measurement, the Pearson correlation coefficient between the absolute errors and the reference angulations is 0.18, inferring low correlations between the degree of fracture and aligning POCUS images. Nevertheless, the small cohort size, i.e., 11 samples, resulted in the coefficient being less significant. Future work will focus on curating more POCUS images and improving image quality with noise suppression.

## Conclusion

We proposed a fully automatic pipeline for boundary segmentation, landmarks detection for POCUS image pairs, and aligning them from different views. The proposed pipeline is designed to replace X-rays for pediatric distal forearm fractures for the first time, and the results demonstrate low errors in terms of distance to reference X-ray images, high accuracy in classifying fracture types, and comparable performance in measuring angulation against reference X-rays, highlighting its effectiveness and potential to assist surgeons.

However, several limitations need to be addressed in future work; for example, the pipeline relies highly on detected landmarks, which can fail in extreme cases with a high amount of noise in POCUS. Moreover, the POCUS image pair is not necessarily captured in the same imaging plane and is often misaligned with the real boundary of the bone due to deviations in the collecting orientation, resulting in ambiguity in aligned results compared to reference X-rays. Similarly, the reference X-rays also suffer from projection issues, causing inaccurate physical scale compared to POCUS images. Future work will focus on collecting more POCUS images to improve the performance in detection and alignment and the robustness against ultrasound noise.

## Supplementary Information

Below is the link to the electronic supplementary material.

Supplementary file 1 (pdf 306 KB)

## References

[1] Larsen AV, Mundbjerg E, Lauritsen JM, Faergemann C (2020) Development of the annual incidence rate of fracture in children 1980–2018: a population-based study of 32,375 fractures. Acta Orthop 91(5):593–597 32500789 10.1080/17453674.2020.1772555 PMC8023904

[2] Beck SM, Schwerk P, Fitze G, Schultz J (2023) Transepiphyseal percutaneous intramedullary kirschner wire (tepik) in diametaphyseal radius fractures (dmrf)-experiences in 59 children. J Pediatr Surg Open 3:100033

[3] Pohl JE, Schwerk P, Mauer R, Hahn G, Beck R, Fitze G, Schultz J (2024) Diagnosis of suspected pediatric distal forearm fractures with point-of-care-ultrasound (pocus) by pediatric orthopedic surgeons after minimal training. BMC Med Imaging 24(1):255 39334059 10.1186/s12880-024-01433-y PMC11428926

[4] Ackermann O, Liedgens P, Eckert K, Chelangattucherry E, Ruelander C, Emmanouilidis I, Ruchholtz S (2010) Ultrasound diagnosis of juvenile forearm fractures. J Med Ultrason 37:123–127 10.1007/s10396-010-0263-x 27278011

[5] Bartoníček J, Naňka O (2024) The true history of the hueter-volkmann law. Int Orthop 48(10):2755–2762 39083236 10.1007/s00264-024-06254-w PMC11422464

[6] Lautman S, Bergerault F, Saidani N, Bonnard C (2002) Roentgenographic measurement of angle between shaft and distal epiphyseal growth plate of radius. J Pediatr Orthop 22(6):751–753 12409901

[7] Hosseinzadeh P, Olson D, Eads R, Jaglowicz A, Goldfarb CA, Riley SA (2018) Radiologic evaluation of the distal radius indices in early and late childhood. Iowa Orthop J 38:137 30104936 PMC6047389

[8] Isensee F, Jaeger PF, Kohl SA, Petersen J, Maier-Hein KH (2021) nnu-net: a self-configuring method for deep learning-based biomedical image segmentation. Nat Methods 18(2):203–211 33288961 10.1038/s41592-020-01008-z

