# Supplementary material for: Automatic ultrasound image alignment for diagnosis of pediatric distal forearm fractures
Source: Int J Comput Assist Radiol Surg. 2025 May 2;20(6):1249–54. doi: 10.1007/s11548-025-03361-w (PMC12167337; doi:10.1007/s11548-025-03361-w)
Supplement: Supplementary file 1 — (pdf 306 KB) [file 11548_2025_3361_MOESM1_ESM.pdf]

Automatic Ultrasound Image Alignment for Diagnosis of Pediatric Distal Forearm Fractures

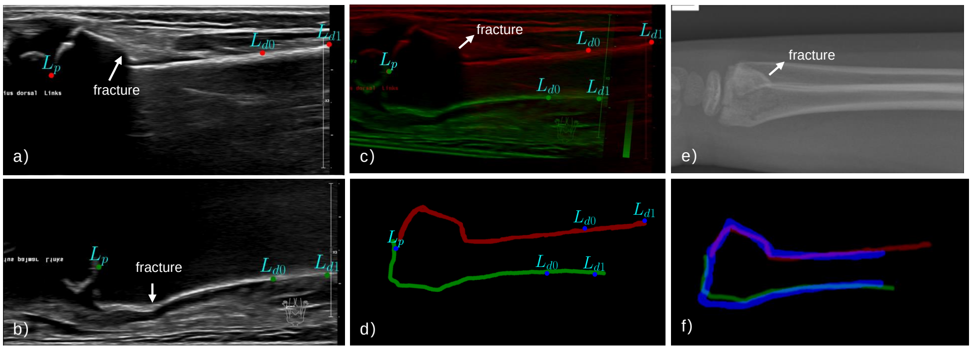

Fig. 1 POCUS and X-ray images of a patient with one greenstick fracture. Obvious discontinuity can be found on the upper boundary in POCUS image, this sample corresponds to Sample 3 in Tab.1 and Tab.2 in the main text. a). A higher level of noise can be located in the bottom right corner of b) with a blurry bone boundary, possibly resulting from imperfect placement of the POCUS probe. As a consequence, this results in an inaccurate boundary segmentation, which can be seen in d), and misplaced landmarks, i.e. L_{d0}andL_{d1}.

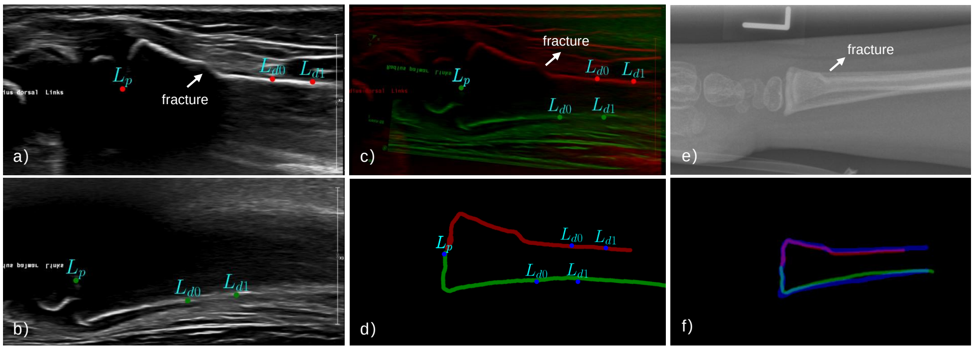

Fig. 2 POCUS and X-ray images with one buckle fracture. A higher level of noise is visible near the bone boundary in b), this did cause erroneous detection of landmarks, proved by the nice alignment between the segmented boundaries from POCUS and X-rays.
